# An Approach to Share Self-Taught Knowledge between Home IoT Devices at the Edge †

**DOI:** 10.3390/s19040833

**Published:** 2019-02-18

**Authors:** Ingook Jang, Donghun Lee, Jinchul Choi, Youngsung Son

**Affiliations:** IoT Research Division, Electronics and Telecommunications Research Institute, Daejeon 34129, Korea; donghun@etri.re.kr (D.L.); spiders22v@etri.re.kr (J.C.); ysson@etri.re.kr (Y.S.)

**Keywords:** Internet of Things, intelligent IoT, smart home, edge computing, knowledge sharing

## Abstract

The traditional Internet of Things (IoT) paradigm has evolved towards intelligent IoT applications which exploit knowledge produced by IoT devices using artificial intelligence techniques. Knowledge sharing between IoT devices is a challenging issue in this trend. In this paper, we propose a Knowledge of Things (KoT) framework which enables sharing self-taught knowledge between IoT devices which require similar or identical knowledge without help from the cloud. The proposed KoT framework allows an IoT device to effectively produce, cumulate, and share its self-taught knowledge with other devices at the edge in the vicinity. This framework can alleviate behavioral repetition in users and computational redundancy in systems in intelligent IoT applications. To demonstrate the feasibility of the proposed concept, we examine a smart home case study and build a prototype of the KoT framework-based smart home system. Experimental results show that the proposed KoT framework reduces the response time to use intelligent IoT devices from a user’s perspective and the power consumption for compuation from a system’s perspective.

## 1. Introduction

Recent advances in the Internet of Things (IoT) have changed the lifestyle of people in various environments in which many of electronic devices around us, such as smart appliances, mobile devices, sensors, and wearables, are connected to the network. Since a wide range of practical applications has absorbed enormous amounts of sensors and devices, the number of IoT devices connected to the Internet has increased more rapidly. More than 5 million new IoT devices are getting connected to the Internet every day and the number of connected devices will reach more than 20 billion by 2020 [1].

To move beyond connectivity, the IoT which employs artificial intelligence (AI) and machine learning (ML) has been envisioned as the next wave in the era of the IoT. Gartner lists *intelligent things* as one of the top 10 strategic technology trends for 2018 [2]. To provide intelligent services to customers, IoT devices need informative models trained from data. In typical IoT systems, model training is performed on high-performance computing entities with multiple CPUs and GPUs, and inference using such generated models is executed on devices. However, on-device learning has been on the rise recently [3,4,5]. IoT devices will be able to produce *self-taught knowledge* by training models locally from their acquisition of sensing data in order to perform real-time inference.

In this trend, the next step is to explore how to share self-taught knowledge between intelligent IoT devices with minimal human intervention. So far, users or systems have repeated the same procedure when using multiple IoT devices for which similar or even identical knowledge is required. This leads to an increase in behavioral repetition in users and computational redundancy in systems. For example, in a smart home, each family member repeatedly participates in training for face recognition whenever an IoT device that provides personalized services (e.g., smart mirrors, smart doorbells, home robots, and other IoT devices) is newly deployed at home. Moreover, multiple devices produce identical knowledge for face recognition and store the duplicate into their own storage spaces. These issues can be more problematic in large-scale IoT applications (e.g., smart building and smart city) due to a large quantity of IoT devices.

To make IoT devices exchange information with each other, cloud computing is a good technical approach and works in a centralized manner. At first glance, the cloud-based service model seems to be an appropriate solution to the aforementioned problems of sharing of self-taught knowledge. However, given the increasing growth rate of the IoT, the cloud may reveal several limitations including scalability, bandwidth, latency, and privacy [6]. Edge computing is a technological effort to alleviate such problems as a complementary partner to cloud computing, which defines a decentralized topology where computing and processing are placed closer to sources of data. Various IoT applications require information processing and content delivery to be shifted from the *cloud to the edge* where the constraints of the cloud can be relaxed [7] (it is also mentioned as one of top 10 technology trends in 2018 [2]).

In this paper, we propose a framework which provides self-taught knowledge sharing between IoT devices at the edge in order to reduce behavioral repetition in users and computational redundancy in systems. This paper builds on our previously published paper [8] and includes a detailed description of the proposed framework. Section 2 presents the related work and Section 3 introduces the proposed *Knowledge of Things* (*KoT*). We give a smart home case study for understanding the KoT in Section 4, and the system implementation and evaluation are provided in Section 5. Finally, Section 6 concludes this paper.

## 2. Related Work

The IoT has been one of the most popular research topics in recent years. The IoT can be described as the convergence of the three different perspectives: *things-oriented*, *Internet-oriented*, and *semantic-oriented* visions [9]. The things-oriented perspective is defined as hardware technologies. The Internet-oriented aspect is characterized by connectivity and interoperability between multiple entities. The semantic-oriented perspective can be defined as machine intelligence to enable devices to produce and process knowledge for automated decision-making. In this section, recent related studies are discussed from these three perspectives of the IoT.

### 2.1. Things-Oriented Perspective

The things-oriented vision can be realized through hardware technologies. The evolution of hardware components and the improvement of the computing power allow resource constraint devices to have higher computation capacity. The progressive increase of computing resources enables end devices to perform not only sensor data gathering but also data analyzing and decision making. Moreover, IoT devices should be capable of producing the necessary knowledge on their own. To meet the increasing demands of autonomous and intelligent things in various IoT applications, the hardware technologies are expected to evolve continuously in the coming decades.

### 2.2. Internet-Oriented Perspective

The Internet-oriented perspective can be derived from the connectivity/interoperability provision, which is represented as IoT standards and platforms for machine-to-machine (M2M) communications. The development of various IoT standards and platforms has focused on providing connectivity and interoperability between heterogeneous devices by abstracting complexities of networked systems in the IoT. Multiple IoT platforms have developed (e.g., *AllJoyn* [10], *IoTivity* [11], *Thread* [12], *Weave* [13], and *FIWARE* [14,15]) and global standards (e.g., oneM2M [16]) have been specified.

**Figure 1:**
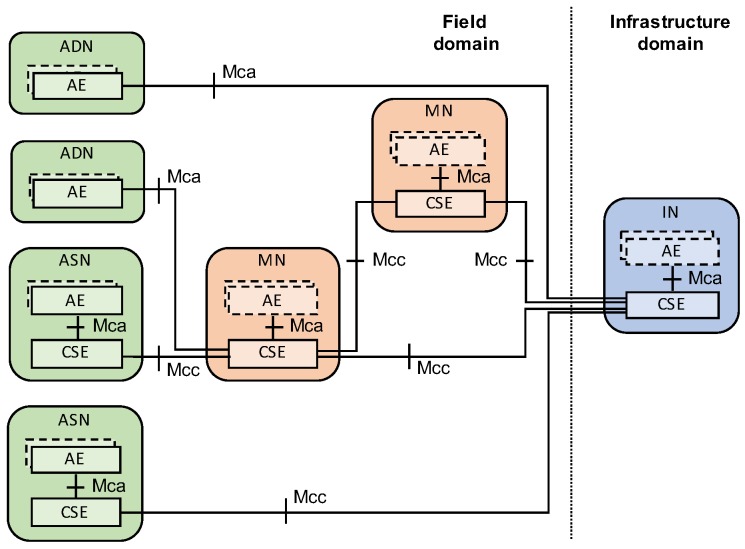
oneM2M functional architecture.

The oneM2M is an international standard led jointly by the eight standard development organizations (Association of Radio Industries and Businesses (ARIB), Japan; Alliance for Telecommunications Industry Solutions (ATIS), United States; China Communications Standards Association (CCSA), China; European Telecommunications Standards Institute (ETSI), Europe; Telecommunications Industry Association (TIA), United States; Telecommunications Standards Development Society (TSDSI), India; Telecommunications Technology Association (TTA), Korea; and Telecommunication Technology Committee (TTC), Japan), established in order to develop a globally applicable common architecture for M2M communications and the Internet of Things. The oneM2M have mainly focused on providing connectivity between devices for data exchange, identification of devices and applications, and interoperability among other IoT platforms. Figure 1 illustrates the oneM2M functional architecture which includes several logical nodes; an infrastructure node (IN), a middle node (MN), an application service node (ASN), and an application dedicated node (ADN). Each node is composed of at least one application entity (AE) or at least one common service entity (CSE). An AE provides application logic to users and a CSE serves common service functions (CSFs) such as registration, device management, data management, discovery, group management, and subscription/notification. The oneM2M employs a RESTful architecture including resource-based data model and CRUD resource operations (CREATE, RETRIEVE, UPDATE, and DELETE). All data and information are stored as resources and accessed through CRUD operations via various communication protocols such as HTTP, MQTT, and CoAP.

As another aspect of the Internet-oriented vision, an IoT system should be developed considering whether the cloud or edge computing is more appropriate to its architecture. In general, most recent IoT architectures have been developed based on the centralized cloud model [17,18,19,20,21]. However, cloud-based IoT systems encounter significant challenges in terms of scalability, bandwidth, and latency. In most cloud-based IoT applications, the quantity and velocity of data produced by IoT devices have increased rapidly and enormous amounts of data are exchanged with the remote data centers of cloud service providers. To efficiently handle the inevitable increases in data, it will be more challenging to resolve the scalability and bandwidth issues of the IoT spaces. Latency should be also considered to support time-sensitive IoT applications. In cloud-based IoT applications, the data travels across the whole network path; sensing, transferring local data, processing in local gateways, delivering via the Internet, and processing in the cloud. The data can experience considerable latency and this problem can be more critical to time-sensitive IoT applications (e.g., disaster management and healthcare) which require real-time responses. Security and privacy concerns also should be considered. Sensors and devices deployed in our vicinity may collect personal and sensitive information from users and store such information in the centralized data centers (e.g., healthcare [21]). Therefore, security and privacy can be potentially violated.

In an effort to tackle the challenges of cloud computing, edge computing has been emerging as a new paradigm for computing and data processing near data sources. Edge computing allows computing and processing to be performed at a level of an edge by placing any computing resources along the path between end-devices and data centers in the cloud. Edge computing significantly reduces the data volume which has to be exchanged with the cloud and thus decreases latency. The flexible architecture of edge computing can provide higher scalability compared to the existing cloud architectures. In addition, if an edge entity is able to make a decision, the IoT devices can continue to work intelligently even if the Internet connection from the edge to the cloud gets lost. Based on these advantages, various applications have employed the edge computing for building their own IoT architectures [22,23,24].

Recently, fog computing is introduced to provide intermediate processing, computation, and networking between the edge and cloud [25,26]. The fog computing seamlessly integrates the two computing paradigms to efficiently use edge devices and cloud resources. This new computing paradigm provides location-aware services to users with a better quality of service (QoS) in terms of latency, real-time response, data traffic control between the edge and cloud in many IoT applications [27,28,29].

### 2.3. Semantic-Oriented Perspective

The last vision of IoT realization is the semantic-oriented perspective which includes data analysis, reasoning, and automated decision-making in order to make IoT devices and applications more intelligent. State-of-the-art AI techniques such as neural networks, evolutionary algorithms, and reinforcement learning are embedded directly to IoT devices for intelligent IoT applications including autonomous vehicle [30], smart factory [31], and smart retail commerce [32]. These applications include enormous IoT devices and they need to be trained individually in order to provide intelligent services. In this situation, if an IoT device can share self-taught knowledge to other devices which require similar or even identical knowledge, user’s behavioral repetition and the overhead cost due to redundant training processes can be reduced significantly.

The Semantic Web is a representative technology for the semantic-oriented perspective, which extends the Web with a resource-based way and uses machine-readable meaning to provide knowledge (information) representation and sharing among heterogeneous machines. The Resource Description Framework (RDF) [33] and Web Ontology Language (OWL) [34] are designed for the Semantic Web and they enable knowledge representation and relationship modeling. The semantic technologies allow machines to facilitate automated knowledge reasoning and decision making. These aspects have led to the integration of the semantic technologies with various research domains such as e-commerce [35,36,37], medical services [38,39,40,41], bioinformatics [42,43,44], and manufacturing systems [45,46,47].

The semantic technologies may help devices to interact with others and share their information intelligently from an IoT perspective. However, only a few research studies have focused on the Semantic Web-based knowledge sharing in IoT environments [48,49]. It is inherently difficult to apply the semantic technologies to IoT applications due to the following limitations: (1) heterogeneity of data models, interfaces, and communication protocols, (2) lack of interoperability of IoT devices, (3) considerable demands in computing resources for reasoning [50]. To spread the semantic technologies over the IoT fields, some challenges such as scalable and heterogeneous data processing, efficient knowledge sharing, and real-time reasoning should be considered [51].

As we summarize the recent trends and challenges of the aforementioned three perspectives of the IoT, it is critical to study how to share knowledge between IoT devices. Our proposed framework based on an IoT platform can support self-taught knowledge sharing between IoT devices in a non-semantic manner at the edge level.

## 3. KoT Framework

In this section, we introduce the concept and design of our proposed KoT framework.

### 3.1. Definitions

We give some definitions required for understanding the proposed framework as follows:

- *Knowledge of Things (KoT)*: We define the Knowledge of Things (KoT) at the conceptual level, which supports sharing of self-taught knowledge between IoT devices. The proposed KoT framework provides management services for the KoT between IoT devices.
- *Self-taught knowledge (STK)*: An STK element is represented as an informative model generated from machine learning techniques which can be performed locally by an IoT device.
- *Contribute and catch operations*: We define two important operations used in the KoT framework. The contribute operation is to push an STK element generated by an STK contributor into a KoT repository which resides in an edge entity in order to store a set of STK elements. An STK catcher performs the catch operation which pulls necessary knowledge from the KoT repository. An IoT device with sufficient computing resources can play both roles of a contributor and a catcher. Otherwise, a resource-constraint device mainly can be a catcher.

### 3.2. Proposed KoT Framework

The proposed KoT framework can be integrated into IoT platforms. The IoT platform provides connectivity between IoT devices and manages information and data as resources and the KoT framework enables IoT devices to produce, cumulate, and share their own self-taught knowledge with each other at the edge level.

Typically, the application layer consumes the data generated from end devices and managed in the IoT platform layer. So far, most of the data generated from end devices are raw sensory data. However, as the computing power of IoT devices increases (e.g., IoT devices embedding GPU units or deep learning processors), the end devices can produce an STK element (i.e., a trained model) for themselves by exploiting machine learning techniques. Therefore, the IoT platform layer should store and manage such STK elements as a new type of resources. The proposed KoT framework provides the *intelligent service functions* (*ISF*s) required to deal with STK elements within the IoT platform.

**Figure 2:**
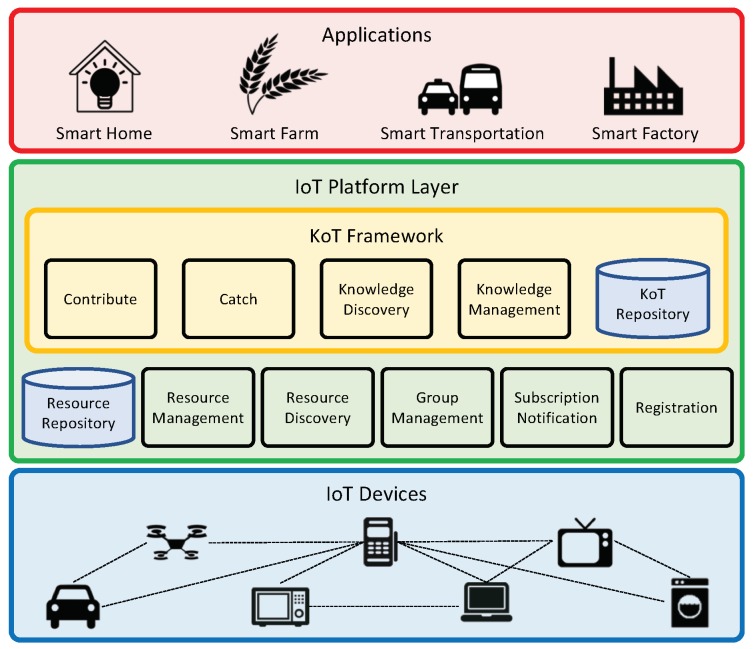
Architecture of the KoT Framework integrated into the IoT platform layer.

Figure 2 illustrates the architecture of the KoT Framework integrated into an IoT platform. The integrated version of the IoT platform and the KoT framework is embedded into each IoT device. The Contribute and Catch ISFs are mutually bidirectional operations which are to store STK elements into the KoT repository and to extract them from the KoT repository, respectively. The contribute function is executed by an STK contributor and the STK element can be stored in both the KoT repository of the edge entity (e.g., home gateway) and the local storage of the IoT device. The goal of the catch function is to allow a new IoT device to possess a well-trained STK element by pulling it from the KoT repository of the edge entity. The extracted STK element is stored in the local storage of a new STK catcher in order to immediately provide an intelligent service to users.

The Knowledge Discovery ISF searches meta-information about an STK element stored as a resource in the KoT repository. An STK catcher sends a query for the information of the necessary knowledge to an edge entity before the catch function. The edge entity responds with a message containing the query result to the STK catcher and then it performs the catch operation if available.

The Knowledge Management ISF is responsible for providing and managing the KoT repository and functions. This ISF should include the capability of converting an STK element into a specified format of a resource and sending a notification about a change in the stored STK element to IoT devices which have an interest in that knowledge.

**Figure 3:**
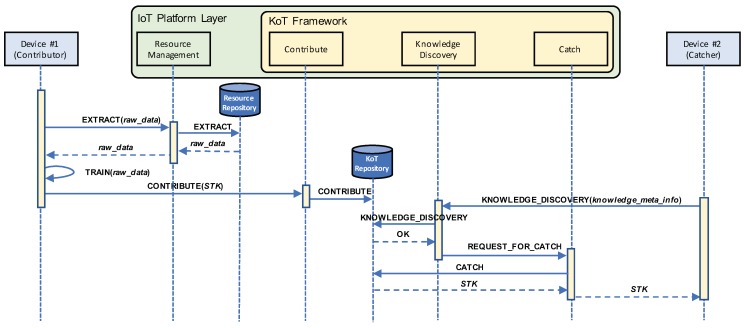
Sequence diagram of sharing of self-taught knowledge between two IoT devices.

Figure 3 shows a sequence diagram of self-taught knowledge sharing between two IoT devices. Device #1 extracts the collected data either from the resource repository of the edge entity or its local storage and trains a model by using machine learning to produce an STK element. In this case, device #1 delivers a CONTRIBUTE message with the generated STK element to the KoT repository of the edge entity (and also stores it in its local storage). Device #1 can provide an intelligent service to users by using the self-taught knowledge stored in its local storage. When a new device needs the identical knowledge, it sends a KNOWLEDGE_DISCOVERY message with meta-information of a necessary STK element in order to CATCH it from the KoT repository of the edge entity. If the discovery succeeds, the found STK element is delivered to device #2 and it can provide an intelligent service to users immediately.

## 4. Smart Home Case Study

In this section, we give a case study of a smart home, which illustrates our vision of the Knowledge of Things.

### 4.1. Use Case Scenarios

We describe some scenarios based on the concept of the Knowledge of Things in a smart home environment which is suitable for an edge-based service model where a user uses a pair of a smart mirror and a smart doorbell. These scenarios show that self-taught knowledge sharing through the KoT framework makes different IoT devices to collaborate with each other autonomously.

#### 4.1.1. Contribute by the Smart Mirror

**Figure 4:**
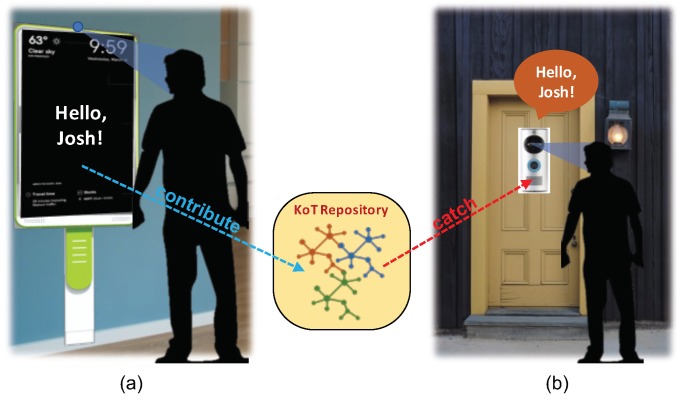
Use case scenario of sharing of self-taught knowledge: face recognition for a smart mirror and a smart doorbell; (**a**) the smart mirror contributes its STK element to a KoT repository and (**b**) the smart doorbell catches the necessary STK element from the KoT repository. (Adapted from [8] with permission of IEEE).

A user deploys a smart mirror in a house, which can provide personalized services for each family member after face recognition. To distinguish faces of family members, the smart mirror has to train the face recognition model during the initial operation. In this case, the smart mirror can contribute its self-taught knowledge for face recognition into the KoT repository, as shown in Figure 4a. When a new member is added, the smart mirror contributes again to update the additional knowledge to the KoT repository.

#### 4.1.2. Catch by the Smart Doorbell

The user deploys another new IoT device; a smart doorbell which can recognize faces of family members and visitors and notify their visits to the house owner’s other device such as a smartphone or a smart mirror. In general, all of the family members should repeatedly participate in the training process for face recognition whenever an IoT device is newly deployed in the house, which has the functionality of face recognition. However, in the proposed KoT concept, the smart doorbell can communicate with an edge entity (e.g., home gateway) and easily catch the necessary STK element from the KoT repository, as shown in Figure 4b. With a help of the KoT framework, family members can use the smart doorbell right after deployment.

#### 4.1.3. Vice Versa

The smart mirror can help the initial operation of the smart doorbell through the KoT framework, and vice versa. The smart doorbell can also contribute its self-taught knowledge about the frequency of visits to the KoT repository. In the case of frequent visitors such as friends, relatives, and neighbors, the smart mirror will announce the arrival of a frequent visitor to in-house members by catching the self-taught knowledge, produced by the smart doorbell, from the KoT repository.

### 4.2. Design Goals

Our proposed smart home system with the smart mirror and the smart doorbell is designed to achieve the following major goals:

1. *Face model training*: The smart mirror should be able to produce a face model by training facial photos of family members through its embedded camera.
2. *Face STK sharing*: The generated face STK should be shared with the smart doorbell via the proposed KoT framework. The overall process should be done at the edge level.
3. *Collaboration*: The smart mirror and the smart doorbell can collaborate by sharing their own generated STK with each other, although each of them can individually provide its service to users.

In addition to these main goals, various other functions can be implemented in the future, including home security, delivery notification, etc. According to the scope of this paper, we have focused on the main goals.

## 5. Implementation and Evaluation

We have implemented the proposed KoT framework over the oneM2M specification. The main reason the oneM2M is chosen is that its standardization is actively being specified as a global standard, and technologies for providing interoperability with other IoT platforms continue to be developed. However, the scope of the oneM2M specification is limited to exchange of information sensed by IoT devices and does not consider processing and sharing knowledge self-taught by intelligent devices. In this section, we demonstrate and evaluate the feasibility by implementing the proposed KoT framework integrated with the oneM2M.

### 5.1. Hardware Components

**Figure 5:**
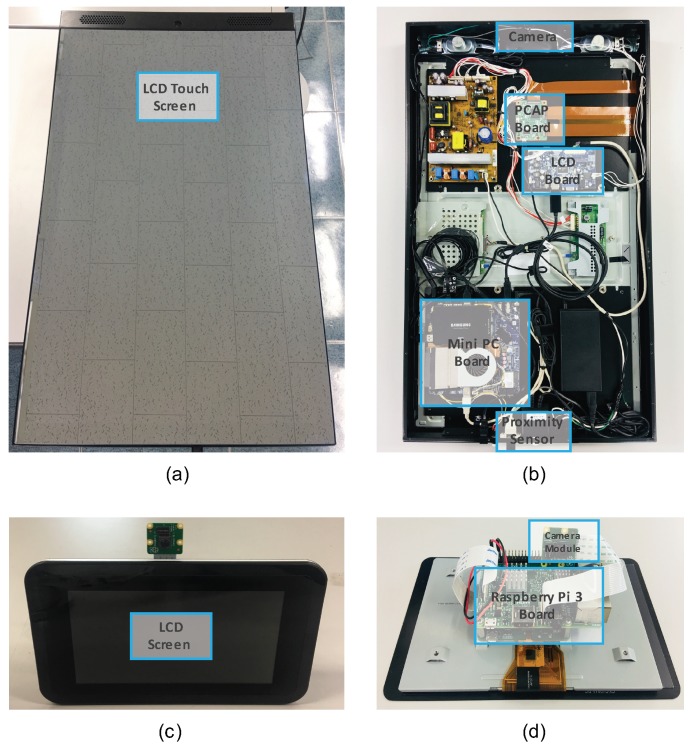
Hardware components of the prototype with a smart mirror and a smart doorbell; (**a**) the external view of the smart mirror, (**b**) the internal view of the smart mirror, (**c**) the external view of the smart doorbell, and (**d**) the internal view of the smart doorbell.

Figure 5 shows the hardware components of our proposed system with the smart mirror and the smart doorbell. All the components are wirelessly connected to an access point. The specific descriptions of the components are listed as follows:

1. *Home gateway*: A home gateway is quite suitable for an edge entity in a smart home environment [7]. All IoT devices should be registered to the home gateway in order to make them aware of each other’s existence. From the oneM2M perspective, the home gateway operates as a middle node (MN) and needs to have sufficient computing power to embody an MN-CSE. The MN-CSE stores all the information related to the edge it manages (registered IoT devices, sensed data, and generated STK elements). The gateway communicates with registered devices through the home network.
2. *Smart mirror*: As one of the end devices, the smart mirror includes an ASN-CSE connected to the MN-CSE of the home gateway through registration. To provide personalized services to users after face recognition, the following items are equipped on the smart mirror:
  (a) *Mainboard*: All underlying components are directly equipped to the mainboard which coordinates with a camera module, a proximity sensor unit, and a touch display to execute computing functions. We use a mini PC board as the mainboard of the smart mirror. The processor (https://ark.intel.com/en/products/88187/Intel-Core-i5-6400T-Processor-6M-Cache-up-to-2-80-GHz-) embedded in this board consumes approximately 35 Watts of power.
  (b) *Camera*: A camera module is used to take facial photos and recognize users from incoming images. We use a webcam connected to the mainboard of the smart mirror via the USB connection.
  (c) *Proximity sensor*: To reduce power consumption, a proximity sensor unit wakes up the smart mirror’s screen only when it senses the presence of someone in front of the smart mirror.
  (d) *User interface hardware (LCD touch display)*: A touch display shows information for users and detects the location of touches as inputs from users. This touch display is controlled by the PCAP (Projected CAPacitive) board.
3. *Smart doorbell*: The smart doorbell also includes an ASN-CSE which is able to communicate with the MN-CSE of the home gateway.
  (a) *Board*: To make the smart doorbell programmable, we use the Raspberry Pi 3 which is one of the most popular open-source hardware modules. This board consumes 5 Watts of power approximately [52].
  (b) *Camera*: The smart doorbell recognizes visitors from the images captured by an embedded camera module.
  (c) *User interface hardware (LCD display)*: A display shows user-friendly information to a visitor, which includes welcome messages and face recognition results.

### 5.2. System Design

**Figure 6:**
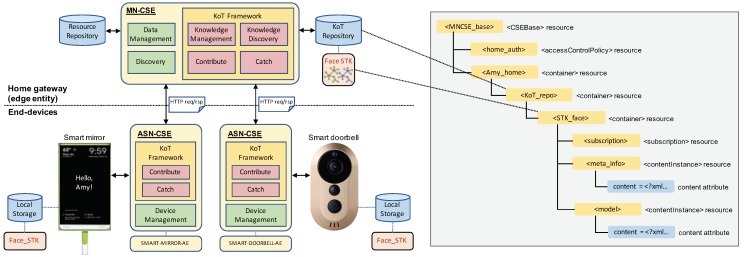
Smart home system design with the smart mirror and the smart doorbell, including oneM2M entities, the KoT framework, and the resource tree of the face STK. (Reproduced from [8] with permission of IEEE).

We have designed the smart home system with the smart mirror and the smart doorbell which operate collaboratively by sharing their self-taught knowledge. Figure 6 illustrates the overall structure of the smart home system with the smart mirror and the smart doorbell. We give some explanations of this structure as follows:

1. *User interface software*: The basic user interface (UI) of the smart mirror is implemented based on MagicMirror2 (https://magicmirror.builders/) which is a popular open-source platform for building a smart mirror. In this platform, small modules that provide specific functions such as calendar, mail, and news can be easily installed into the platform and anyone can write own modules. We adopted this open-source software for the UI of the smart doorbell as well as that of the smart mirror.
2. *Face recognition*: For training and using a face model in the smart mirror and smart doorbell, we use a module using Local Binary Pattern Histogram (LBPH)-based face recognizer (https://docs.opencv.org/2.4/modules/contrib/doc/facerec/facerec_tutorial.html) in OpenCV. The LBPH is well suited for use in the smart mirror and the smart doorbell (i.e., the mini PC board and the Raspberry Pi, respectively). The LBPH algorithm is more computationally efficient than other methods such as Eigen-face and Fisher-face that the OpenCV library provides [53], and this method is robust in terms of illumination changes [54,55]. This method can be applied to learning-based Support Vector Machine (SVM) [56] to improve performance, which is the most widely used classification method.
3. *KoT framework on oneM2M entities*: The KoT framework which resides in each CSE allows IoT devices to easily contribute and catch STK elements to and from the KoT repository of the home gateway. CSEs exchange their messages via HTTP requests/responses. An STK contributor embodies the meta-information and contents of the produced knowledge into the body of an HTTP request message and then sends it to the home gateway. An STK catcher receives a corresponding HTTP response message including the necessary knowledge from the home gateway after discovery. In our smart home system, the smart mirror contributes its face STK element to MN-CSE’s KoT repository through the messaging interface between ASN-CSE of itself and MN-CSE of the home gateway. The smart doorbell catches the face STK element from the KoT repository in MN-CSE. The caught STK element is stored in the local storage of the smart doorbell for face recognition.
4. *Face STK as a resource tree*: We have adopted a data model based on the oneM2M architecture, so all information is designed and represented as resources. <MNCSE_base> is the structural root which stores information about the MN-CSE itself of the home gateway and <home_auth> represents privileges to access descendant resources of the <*someone*_home> container resource. <KoT_repo> contains the <STK_face> resource which consists of <subscription>, <meta_info>, and <model> resources. <subscription> is used to send notifications about changes of the face STK to the related IoT devices. <meta_info> and <model> are contentInstance resources which are able to store actual data in their own content attribute. <meta_info> contains all the information for knowledge discovery in an XML (Extensible Markup Language) format, including *contributor_ID, content_type, timestamp, compatibility*, etc. and <model> keeps the actual trained model in its content attribute. The set of STK elements in the KoT repository ($R^{KoT}$) satisfies the following condition: $R^{KoT}={⋃}_{i\in D}STK^{i}$, where *D* denotes a set of IoT devices involved in a system. Our smart home system simply holds $R^{KoT}=STK^{sm}\cup STK^{sd}$ with the smart mirror ($STK^{sm}$) and the smart doorbell ($STK^{sd}$).

### 5.3. Evaluation

**Figure 7:**
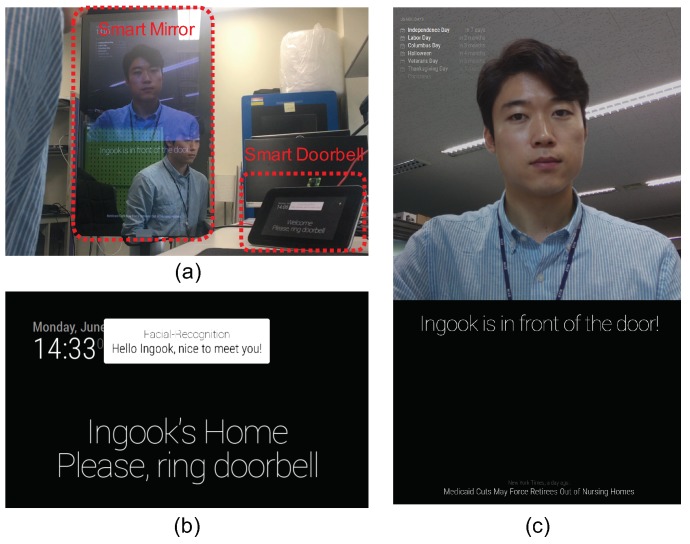
Prototype of our smart home system with a smart mirror and a smart doorbell; (**a**) the smart doorbell notifies someone’s visit to in-house members through the smart mirror, and (**b**) the screen of the smart doorbell and (**c**) that of the smart mirror after face recognition are shown. (Adapted from [8] with permission of IEEE).

Figure 7 shows the operation of the prototype of our smart home system with two IoT devices. Four participants are involved in generating a facial model and the smart mirror trains with one hundred images of each person’s face taken from its camera module. To acquire accurate results, we register only a person as an authorized user who can access our smart home system. We assume that the face STK is produced by the smart mirror and consumed by the smart doorbell because the smart mirror has better computing resources than those of the smart doorbell. After training a model, the smart mirror contributes its face STK element into the KoT repository of the home gateway, and then the smart doorbell catches the face STK element stored in the KoT repository. When a visitor arrives in front of the smart doorbell, it recognizes the visitor’s face by using the face STK element and notifies someone’s visit to in-house members through the screen of the smart mirror.

To evaluate the performance of our system, we compare four variants of our prototype: *smart mirror (SM) standalone*, *smart doorbell (SD) standalone*, *smart mirror (SM) with STK contributing*, and *smart doorbell (SD) with STK catching*. Initial operation of each variant is composed of a combination of five steps including $S^{1}$: data gathering, $S^{2}$: training a model, $S^{3}$: contributing, $S^{4}$: catching, and $S^{5}$: reasoning. We evaluate the response time of each step of four variants and the power consumption according to the number of devices. The measured time includes the total time required to respond to users, i.e., sending an HTTP request, accessing to the KoT repository, internal computation, transferring the actual model, receiving a corresponding HTTP response, and displaying to users. Measuring the response time and the power consumption provide good insights into how much the performance improvement can be achieved in terms of behavioral repetition in users and computational redundancy in systems.

Table 1 shows the results of the time spent on five steps of four variants. The SD standalone consumes tremendous time to take facial photos and train a face model due to lack of computing power, compared to the SM standalone. If the smart mirror based on the KoT framework (the SM w/contributing) contribute its face STK element, the smart doorbell catching this face STK (the SD w/catching) can omit two steps ($S^{1}$: taking photos and $S^{2}$: training a model). Omitting the step of taking photos and that of training a model can reduce behavioral repetition in users and computational redundancy in systems, respectively.

**Figure 8:**
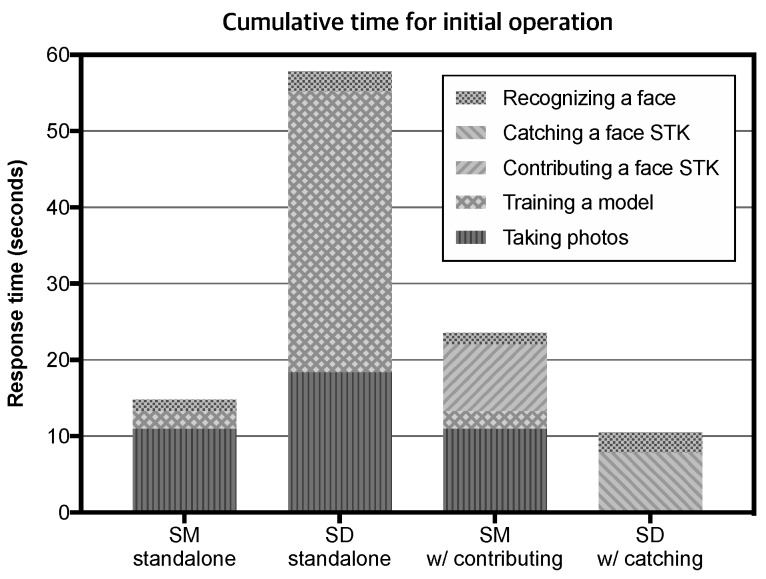
Comparison of initial operation time between four variants of our prototype; smart mirror (SM) standalone, smart doorbell (SD) standalone, smart mirror (SM) with STK contributing, and smart doorbell (SD) with STK catching.

Figure 8 shows the results of cumulative time for the initial operation of four variants. As shown in this figure, although the SM with contributing the face STK consumes more response time than the SM standalone, the SD with catching the face STK can initialize its operation approximately 5.5 times faster than the SD standalone. The KoT framework helps for the SD to eliminate data gathering and training steps, although it consumes time to catch the face STK for initial operation.

**Figure 9:**
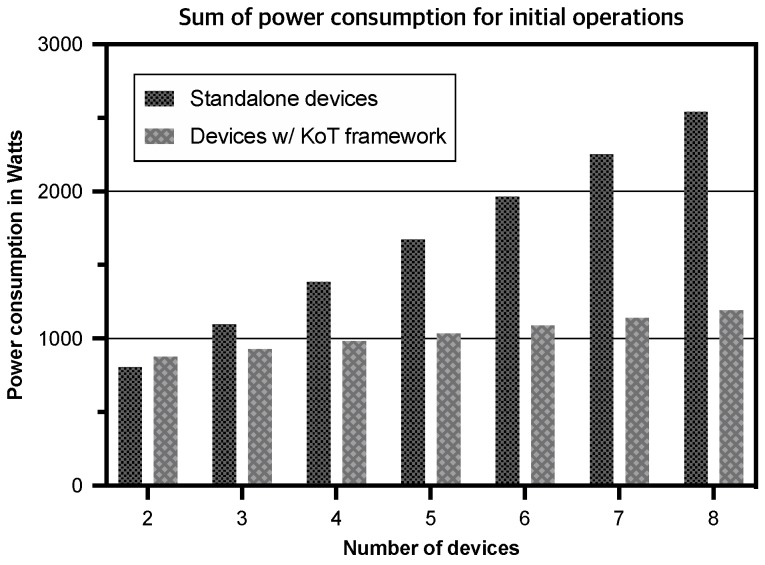
Comparison of the sum of power consumption for initial operations.

Figure 9 illustrates the results of the sum of power consumed by multiple devices. The x-axis represents the number of devices which comprises a smart mirror and (N−1) devices which have a similar computing power with a smart doorbell. The total amount of power consumed by *N* devices is given as:

(1) $$C_{total}=\sum_{j=1}^{N}\sum_{i=1}^{5}P_{j}^{i}T_{j}^{i}$$

where $P_{j}^{i}$ and $T_{j}^{i}$ are the average power consumption (Watt) and the response time (second) of the *j*th device at step $S^{i}$, respectively. We assume that each device uniformly consumes power in each step (the average power consumption of each device is presented in Section 5.1). Although the KoT framework consumes slightly more power when the number of devices is 2, the total amount of power consumption of standalone devices increases more rapidly than that of devices with the KoT framework as the number of devices increases. This result indicates the proposed KoT framework can significantly reduce computational redundancy in larger-scale smart home systems. Therefore, in our case study, we conclude that the proposed KoT framework can reduce the overall response time and the power consumption of our smart home system at the edge, where multiple IoT devices require the similar or even identical self-taught knowledge.

## 6. Conclusions and Further Work

In this paper, we propose a Knowledge of Things (KoT) framework which enables sharing self-taught knowledge between IoT devices which require similar or identical knowledge at the edge. The proposed KoT framework allows IoT devices to effectively share its self-taught knowledge with other devices in the vicinity. We have developed the prototype of the KoT framework-based smart home system. This system consists of a smart mirror and a smart doorbell which require the same knowledge for face recognition. The smart mirror shares its self-taught face STK with the smart doorbell by using the KoT framework, so the smart doorbell can avoid additional user participation and a redundant training process. The results of the experiments have shown that the KoT framework reduces the response time to use intelligent IoT devices from a user’s perspective and the power consumption for computation from a system’s perspective. Therefore, we conclude that this framework alleviates behavioral repetition in users and computational redundancy in systems in intelligent IoT applications.

However, current research stays at the level of the conceptual framework and there are still many studies to be performed. The following studies are required for deployment into practical and large-scale IoT environments, including STK standardization, meta-information specification for knowledge discovery and processing, prioritized analysis of device requirements for performance and capabilities. To improve knowledge interoperability between intelligent IoT devices, standardization of STK and meta-information should be performed as fundamental issues specified by a global standardization organization. Moreover, although different IoT devices belong to the same application, they may require different performance metrics such as accuracy, latency, computation cost, and communication overhead. Therefore, prioritized analysis of performance requirements needs to be conducted depending on what situation each device is placed. This study has to be considered together with device management mechanisms in order to enable an IoT device to understand its capabilities and preferred performance metric according to its applications, situation, and capacity. 

## Figures and Tables

**Table 1:** Measurement of the time spent on each step (seconds).

	SM Standalone	SD Standalone	SM w/Contributing	SD w/Catching
$S^{1}$: Taking photos	10.98	18.34	10.98	−
$S^{2}$: Training a model	2.27	36.82	2.27	−
$S^{3}$: Contributing a face STK	−	−	8.78	−
$S^{4}$: Catching a face STK	−	−	−	7.82
$S^{5}$: Recognizing a face	1.54	2.68	1.54	2.68
